# Epidemiological profile and risk factors of acute appendicitis in India

**DOI:** 10.6026/973206300222376

**Published:** 2026-04-30

**Authors:** Yogesh Kailasia, Siddharth Jain, Ashutosh Garg

**Affiliations:** 1 Department of Surgery, Shyam Shah Medical College and Associated Hospitals, Rewa, Madhya Pradesh, India; 2 Department of Anesthesiology, Shyam Shah Medical College and Associated Hospitals, Rewa, Madhya Pradesh, India

**Keywords:** Acute appendicitis, epidemiology, risk factors, tertiary care, clinical profile

## Abstract

Acute appendicitis is the most common surgical emergency worldwide, with different epidemiological patterns determined by demographic and lifestyle factors. Therefore, it is of interest to establish the epidemiological profile and associated risk factors for acute appendicitis in a tertiary care hospital setting. A prospective observational study was carried out on clinically and radiologically verified cases, with demographic, clinical, laboratory and surgical characteristics examined using acceptable statistical methods. Among 450 patients, the majorities were young males and higher body mass index and smoking were significantly associated with complicated appendicitis (p < 0.05). Thus, we show the epidemiological profile and associated risk factors for acute appendicitis. The significant role of higher body mass index and smoking in complicating the condition for early identification and management strategies is reported.

## Background:

Acute appendicitis is one of the most prevalent surgical emergencies worldwide, posing a considerable healthcare burden [1]. Global epidemiological analyses have revealed geographical differences in incidence and outcomes, indicating socioeconomic and healthcare inequities [2]. To avoid complications, current management guidelines stress early diagnosis and immediate surgical intervention [3]. Delayed presentation and in-hospital treatment have been substantially linked to an increased risk of perforation and intra-abdominal complications [4]. Epidemiological studies consistently show a peak occurrence in the second and third decades of life, with a small male predominance [5]. Emerging evidence suggests that metabolic and lifestyle factors may influence disease severity. Obesity has been linked to increased risks of complicated appendicitis and postoperative morbidity [6]. Similarly, smoking has been linked to an increased inflammatory response and poor clinical outcomes [7]. Inflammatory biomarkers such as total leukocyte count and C-reactive protein (CRP) have been shown to be useful in predicting complicated appendicitis and guiding early therapeutic decisions [8]. Furthermore, hospital-based cohort analyses emphasize the need of identifying modifiable risk variables for reducing complication burden in tertiary care settings [9]. Therefore, it is of interest to describe the impact of metabolic and lifestyle factors, such as obesity and smoking, on the severity of acute appendicitis, as identifying these modifiable risk factors can help reduce complications and improve patient outcomes in India.

## Materials and Methods:

This prospective observational study took place from January 2023 to December 2024 at the Tertiary Care Hospital's Department of General Surgery. All patients with clinical characteristics suggestive of acute appendicitis were evaluated and enrolled after providing informed consent. The Institutional Review Board provided ethical approval.

## Inclusion criteria:

[1] Patients aged ≥12 years presenting with clinical diagnosis of acute appendicitis.

[2] Confirmed diagnosis by imaging (Ultrasound/CT) or intraoperative findings.

[3] Patients consenting for study participation and follow-up.

## Exclusion criteria:

[1] Patients with previous abdominal surgery that could alter clinical presentation.

[2] History of chronic gastrointestinal diseases (Crohn's, ulcerative colitis).

[3] Incidental appendectomies without clinical appendicitis.

[4] Pregnant women, due to altered physiological presentation.

## Data collection:

Demographic information, clinical symptoms, duration of symptoms, BMI, smoking status, laboratory results (WBC, CRP), imaging findings and surgical details were all documented.

## Definitions:

Complicated appendicitis was defined as appendiceal perforation, abscess or gangrene confirmed intra-operatively. BMI classification follows WHO guidelines for overweight and obesity.

## Statistical analysis:

The data was analyzed using SPSS version 26 (IBM, USA). Continuous variables were presented as mean ± SD, whereas categorical variables were represented as frequencies and percentages. Univariate analysis was performed using the chi-squared test and the t-test. Multivariate logistic regression revealed independent risk variables. A p-value of <0.05 was judged statistically significant.

## Results:

A total of 450 patients were included in the study. The mean age was 28.4 ± 12.3 years, with a male predominance (M: F = 1.7:1). The overall perforation rate was 18.9% Table 1 (see PDF). As shown in Table 2 (see PDF), laboratory parameters, including white blood cell count (WBC) and C-reactive protein (CRP), were significantly elevated in patients with complicated appendicitis compared to those with uncomplicated appendicitis. Table 3 (see PDF) presents the sensitivity of imaging modalities used in the diagnosis, with CT scan showing higher sensitivity (95.8%) compared to ultrasound (85.2%). Table 4 (see PDF) outlines the operative findings, revealing that 66.9% of patients had an inflamed appendix, while 18.9% had perforated appendicitis. The age distribution of acute appendicitis cases is depicted in Figure 1 (see PDF), with the highest incidence observed in younger adults. Additionally, Figure 2 (see PDF) highlights the relationship between risk factors, such as BMI and smoking and complication status, demonstrating a significant correlation between these factors and complicated appendicitis.

## Discussion:

The current study's proportion of complicated appendicitis (18.9%) is consistent with previously reported tertiary care cohort studies, in which advanced disease at presentation continues to represent a significant clinical burden [10]. There was a definite peak incidence in the second and third decades of life, with male preponderance, which is consistent with documented epidemiological trends for acute appendicitis [2]. Higher BMI (≥25 kg/m^2^) was found to be significantly associated with complicated appendicitis. Obesity is increasingly recognized as a risk factor for disease severity, probably due to an increased inflammatory response and diagnostic problems in overweight people [11]. Figure 2 (see PDF) shows a disproportionately greater complication rate among patients with high BMI, supporting the statistical findings. Smoking was also strongly associated with complicated appendicitis in our cohort. Smoking-induced systemic inflammatory activation and impaired immune response may contribute to disease progression and increased severity at presentation [6]. The graphical comparison in Figure 2 (see PDF) shows that smokers have a markedly higher proportion of complicated cases than non-smokers. Laboratory parameters aided in severity categorization. Patients with complicated appendicitis had considerably increased leukocyte counts and CRP levels. Previous research has established the predictive significance of inflammatory indicators in detecting perforation and abscess formation [12]. Furthermore, elevated inflammatory condition at admission has been linked to an increased risk of postoperative intra-abdominal abscess formation after appendectomy [13]. These findings highlight the need of incorporating demographic information, modifiable lifestyle factors and inflammatory biomarkers into early clinical assessments to reduce complication rates and enhance surgical outcomes in tertiary care facilities.

## Conclusion:

Acute appendicitis in this tertiary-care population predominantly affected young adults, with a marked male predominance. Higher body mass index, smoking, elevated leukocyte count and raised C-reactive protein were significantly associated with complicated appendicitis and may help identify patients at increased risk. Early recognition of these high-risk individuals through combined assessment of demographic, lifestyle, and inflammatory factors may enable timely intervention and improve clinical outcomes.
